# “Heterotopic ossification after total hip arthroplasty through direct anterior approach without a dedicated orthopaedic table or direct lateral approach: a quasi-randomized single-center study”

**DOI:** 10.1007/s00402-024-05510-3

**Published:** 2024-09-23

**Authors:** Raffaele Iorio, Matteo Romano Cantagalli, Edoardo Viglietta, Federico Corsetti, Yuri Gugliotta, Leonardo Previ, Salvatore Gagliardo, Simone Fenucci, Nicola Maffulli

**Affiliations:** Orthopaedic Unit, S. Andrea Hospital, University of Rome “La Sapienza”, Via di Grottarossa 1035, Rome, Italy

**Keywords:** HO, THA, Heterotopic ossifications, DAA, Minimally-ivasive

## Abstract

**Introduction:**

Heterotopic ossifications (HO) are common after total hip arthroplasty (THA). The invasiveness of surgical approaches plays a relevant role in HO development. The aims of this study were to assess the development of HO 6 months after THA through direct lateral approach (DLA) or direct anterior approach (DAA) without a dedicated orthopaedic table and to assess the clinical impact of HO.

**Methods:**

This is a single-center IRB-approved, quasi-randomized prospective cohort, observational imaging study. Fifty patients underwent primary THA through DLA and 50 through DAA. Age, sex, BMI and side of the affected hip were collected. At the 6 post-operative month the Harris Hip Score (HHS) and the presence of HO (scored through the Brooker classification system) were assessed.

**Results:**

There was no significant difference in the demographic data between groups. Operative time was significantly higher in the DAA group (72 ± 10 min vs. 58 ± 8 min: *p* < 0.03). At 6 post-operative months the incidence of HO was 14% in the DAA group and 32% in the DLA group (*p* = 0.02). Severe HO (Brooker 3–4) were significantly more common in the DLA group (*p* = 0.04). There was no significant difference in the HHS of patients with HO between the DAA and DLA groups. There was no association between poorer clinical outcomes and the severity of HO.

**Conclusion:**

The DAA without a dedicated orthopaedic table is associated with a significant lower incidence of HO than the DLA 6 months after elective THA. Except for the surgical approach, no other factors correlated with the occurrence of HO. Even though a lower HHS was found with severe HO, the correlation between severity of HO and clinical outcomes did not reach statistical significance.

**Supplementary Information:**

The online version contains supplementary material available at 10.1007/s00402-024-05510-3.

## Introduction

Heterotopic ossifications (HO) consist of ectopic production of mature trabecular bone within soft tissues. HO can occur after traumatic injuries, brain injuries or tissue damage after surgery [1–3] and are common after total hip arthroplasty (THA), with an incidence up to 30% [4]. Often asymptomatic, HO can affect post-operative functional outcomes, causing pain and reduced range of motion (ROM) [1, 5–7].

There is a genetic predisposition to HO development [8], but extrinsic risk factors play an important role. Among these, the surgical approach is the most relevant [9, 10]. The direct anterior approach (DAA) is considered a muscle sparing approach [11, 12]. Conversely, the direct lateral approach (DLA) involves detachment and then suture of gluteus medius, with obvious muscle damage. Some articles investigated the invasiveness of DAA through measurement of serum biomarkers [13–15] or imaging [16, 17]. Post-operative HO formation may represent a direct consequence of the invasiveness of different surgical approaches. A dedicated orthopaedic table may allow surgeons to reduce the soft tissue trauma during the DAA even though a may impair direct assessment of the leg length or implant stability.

The aim of this study was to assess the development of HO 6 months after THA comparing the DAA without a dedicated orthopaedic table and the DLA. The hypothesis was that the DLA presents a higher incidence of HO than the DAA even when the DAA is performed without a dedicated orthopaedic table. Furthermore, the clinical impact of HO with both approaches was investigated.

## Methods

This is a single-center IRB-approved, quasi-randomized prospective cohort, observational imaging study. A consecutive series of 100 patients with end stage hip osteoarthritis and who had undergone primary THA between January 2022 and April 2023 was enrolled in the study.

Exclusion criteria included: (1) previous hip fracture or secondary hip osteoarthritis; (2) previous hip/pelvis muscular trauma; (3) previous operations on the affected lower limb; 3) intra-operative use of bone grafting; (4) diagnosis of genetic muscular pathology, chondrocalcinosis, inflammatory arthropathy, or autoimmune disease; (5) any allergy which could affect the peri-operative protocol of medications.

After thorough information about advantages and disadvantages of the different surgical approaches, patients who gave written consent to participate to the study were divided in two groups: 50 patients received THA through a DAA without an orthopaedic table and 50 patients through a DLA. For logistical and internal organizational issues, it was not possible to undertake a randomized controlled trial. Therefore, patients were allocated to undergo a DAA on Monday and Wednesday, and a DLA on Tuesday and Thursday. All the procedures were performed by the same fully trained senior surgeon fully conversant with both approaches. All patients gave written informed consent to be enrolled in this prospective quasi-randomized study.

**Fig. 1:**
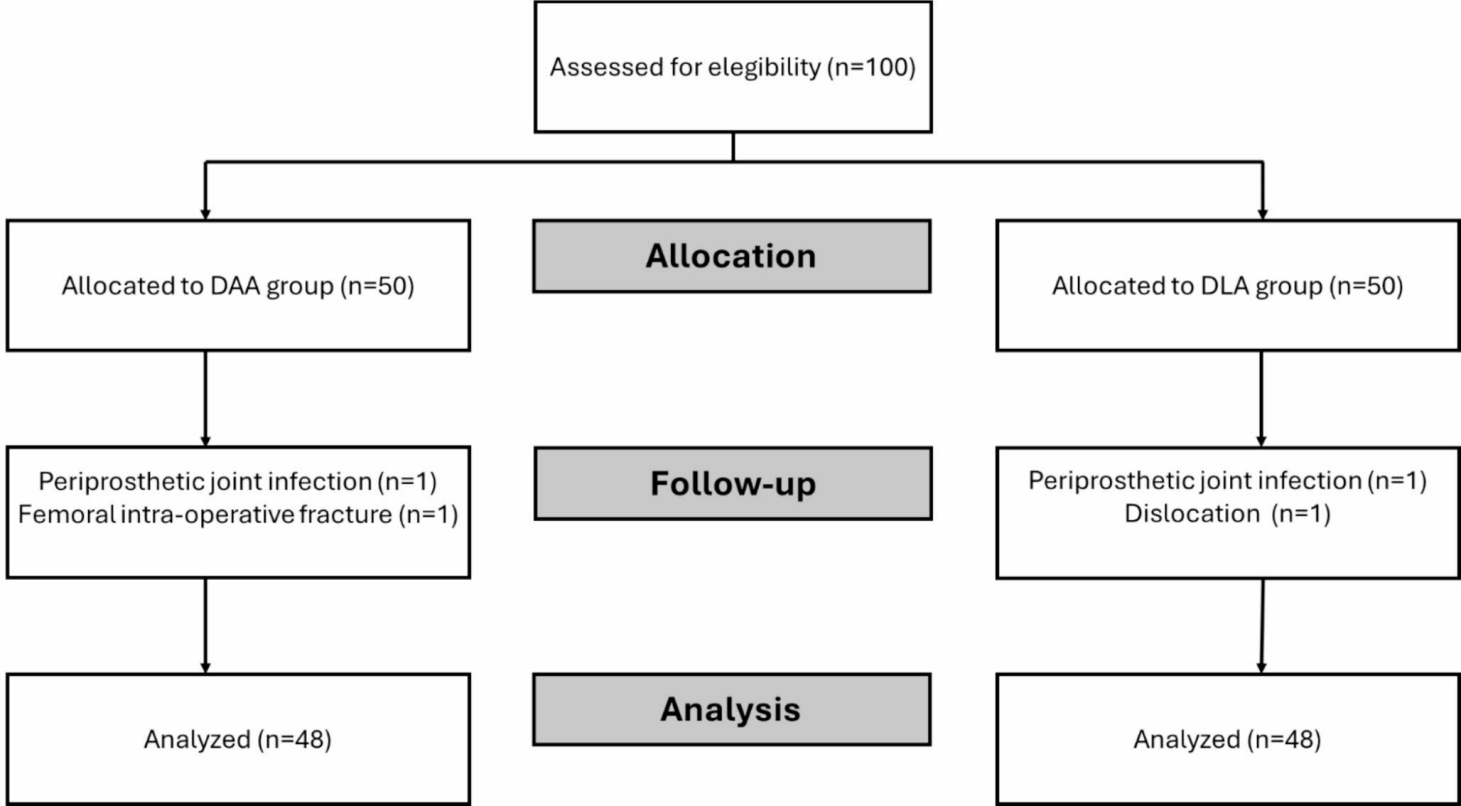
Enrollement

Two patients (one for each group) developed periprosthetic joint infection during the follow-up period. One patient in the DLA group suffered from dislocation 2 months post-operatively and was treated conservatively. In the DAA group, one femoral intra-operative fracture was treated with cerclage without changing the femoral stem. These four patients were excluded from the analysis. Eventually, 48 patients were included in the DAA and 48 in the DLA groups (Fig. 1).

The modified Smith-Peterson DAA was used in the DAA group. Patients were positioned supine on a standard orthopedic table. An 8–10 cm skin incision was performed at the middle third of the junction between the greater trochanter and the anterior iliac spine extending distally towards the fibula head. The interval between the tensor fasciae latae (TFL) and sartorius was developed dissecting the fascia over the TFL to its edge. The ascending branches of the lateral circumflex artery were carefully coagulated, and the lateral femoral cutaneous nerve protected, but not visualized. The plane between rectus femoris and gluteus medius was developed. Hohmann retractors were placed superiorly behind the femoral neck and posteriorly to the greater trochanter, and the anterior capsule was excised to expose the joint. The osteotomy of the femoral neck and the removal of the femoral head were performed taking care to protect the surrounding soft tissues. Acetabular reaming and femoral preparation were performed in the standard fashion.

The modified Hardinge DLA was used in the DLA group. Patients were positioned in a lateral decubitus position on a standard orthopedic table. The skin incision started at the apex of the greater trochanter and extended distally for 8–10 cm in line with the femur. The fascia lata and the trochanteric bursa were incised to expose the glutus medius and vastus lateralis muscle borders. The anterior portion of the gluteus medius tendon was lifted off the greater trochanter and then re-sutured through absorbable Vycril 2 transosseus sutures at the end of the procedure. The vastus lateralis muscle was not sectioned. The hip was dislocated after an anterolateral capsulotomy. Femoral neck osteotomy, head removal, acetabular and femoral preparation were performed in the usual fashion.

For both the surgical approaches, irrigation with pulsating lavage and normosaline solution was used to clean the tissues from residual bone fragments. All patients received antibiotic prophylaxis with 2 g intravenous cefazolin at least 5 min before skin incision. All patients received dexamethasone (8 mg) as part of the anesthetic protocol before surgery. All patients received spinal anesthesia and post-operatively tramadol 20 mg + metoclopramide 10 mg + ketorolac 30 mg in a 24-hour continuous infusion. Absorbable sutures were used for the closure of all the tissues. Surgical drainage was not used. Patients were discharged on the second or third post-operative day with prescription of oral acetaminophen and oral tramadol/oral ketorolac as needed. Low-molecular-weight heparin was administered for thromboembolic prophylaxis for the first 4 post-operative weeks. No patient had prophylactic radiation therapy before or after surgery. In both groups patients did not receive any prophylactic drugs. Several studies investigate the role of anti-inflammatory drugs to prevent HO development. In order to eliminate any confounding factors and to assess the role of the surgical approach on the HO development the Authors decided not to use any prophylactic drugs. All patients were allowed to weight bearing as tolerated from the first-operative day and ROM recovery exercises without any restriction.

Demographic data (age, sex, BMI and side of the affected hip) were collected. Clinical evaluation was performed through the Harris Hip Score (HHS) pre- and post-operatively. HHS was recorded as excellent, good, fair or bad if the score was 100 − 90, 90 − 80, 80 − 70, or < 70, respectively. Radiographic evaluation assessed the presence of HO on anteroposterior pelvis and lateral hip radiographs at 6 months after surgery. The HO grade was defined according to the Brooker classification system. An independent observer, not involved in the surgery and blinded with regard to the surgical approach, collected clinical and imaging data.

Continuously and normally distributed variables were expressed as medians and standard deviations and compared using the Student t test. Non-normally distributed variables were reported as medians and Q1 and Q3 quartiles, and were compared using the Mann-Whitney test. Categorical variables were reported as frequencies and percentages and the Chi-square test and Fisher’s exact test were used. Association between development of HO and age, sex, BMI, pre-operative HHS, operative time and approach was evaluated calculating the Pearson correlation coefficient and linear regression. Statistical analyses were performed using Systat software. Statistical significance was defined as *p* < 0.05.

## Results

There were no statistically significant differences in demographic data between the two groups. Operative time was significantly longer (*p* < 0.03) in the DAA group (72 ± 10 min) compared to the DLA group (58 ± 8 min) (Table 1).

**Table 1:** Demographic data comparison of study groups

Factor	DAA group = 48 Mean/Count	DLA group = 48 Mean/Count	*p*-value
Age (years)	69.4	71.6	$\:p>0.05$
Gender			
Female	25 (52%)	27 (56.2%)	$\:p>0.05$
Male	23 (48%)	21 (43.8%)	
BMI (kg/m^2^)	31	30	$\:p>0.05$
Laterality			
Left	26 (54%)	23 (48%)	$\:p>0.05$
Right	22 (46%)	25 (52%)	
Surgery time (minutes)	72 ± 10	58 ± 8	$\:p<0.03$

**Fig. 2:**
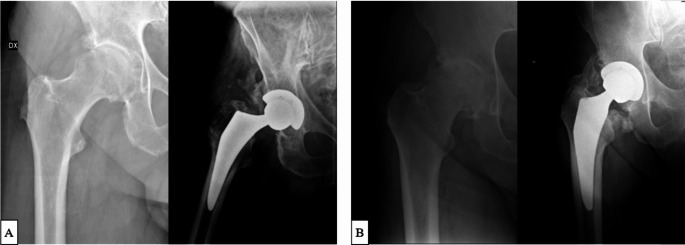
Pre-operative and 6 months post-operative X-rays. **(A)** Brooker 3 HO after DAA approach. **(B)** Brooker 4 HO after DLA approach

**Fig. 3:**
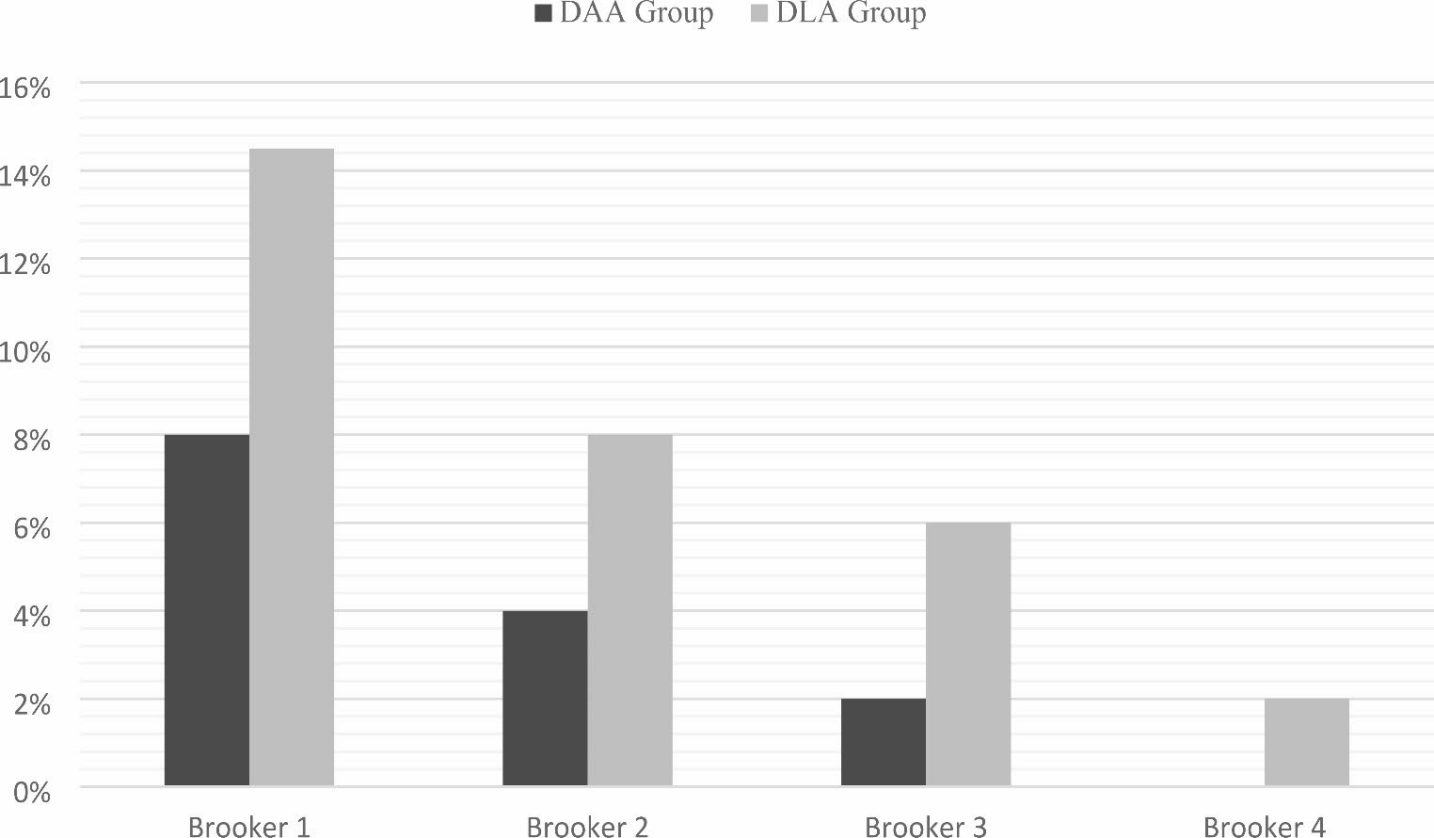
Distribution of HO Brooker grades in DAA and DLA groups

At 6 post-operative months 7 patients (14.5%) in the DAA group and 16 (33%) in the DLA group demonstrated radiographic evidence of HO (*p* = 0.02). The overall incidence of HO was 24%. According to the Brooker classification for the severity of HO, in the DAA group a grade 1 HO was present in 4 patients (8%), a grade 2 in 2 patients (4%), and a grade 3 in 1 patient (2%). No grade 4 were found. In the DLA group, a grade 1 HO was present in 7 patients (14.5%), a grade 2 in 4 patients (8%), and a grade 3 in 3 patients (6%), a grade 4 in 1 patient (2%) (Fig. 2). Comparing DAA and DLA according to the severity of HO development, a significantly higher incidence of severe HO (Brooker 3–4) was found in the DLA group (*p* = 0.04) (Fig. 3).

The HHS showed that in the DAA group 5 patients with HO (70%) had excellent result (HHS > 90) and 2 patients (30%) had good result (HHS 80–89). In the DLA group, 8 patients with HO (50%) had excellent result (HHS > 90), 6 patients (36,7%) had good result (HHS 80–89), and 2 patients (13,3%) experienced fair result (HHS 70–79). No statistically significant differences were found comparing the HHS of patients between the DAA and DLA groups (*p* > 0.05). No statistically significant differences were found comparing the HHS of patients with HO between the DAA and DLA groups (*p* > 0.05). Even though a lower HHS was present in severe HO, there was no evidence of a statistically significant association between poorer clinical outcomes and severity of HO (*p* > 0.05).

No evidence of a statistically significant association was found between HO development and age, sex, BMI, pre-operative HHS, or operative time. Only the surgical approach was statistically associated with differences in the rate of HO occurrence (Table 2).

**Table:** Table 2

Variable	No HO (*n* = 73) Mean/Count	HO (*n* = 23) Mean/Count	*p*-value
Age (years)	68.8	69.3	$\:p>0.05$
Gender			
Female	40 (54.8%)	12 (52.2%)	$\:p>0.05$
Male	33 (45.2%)	11 (47.8%)	
BMI (kg/m^2^)	29.5	31,8	$\:p>0.05$
Pre-operative HHS	89.8	82.5	$\:p>0.05$
Surgical approach			
DAA	41 (85.5%)	7 (14.5%)	$\:p=0.02$
DLA	32 (67%)	16 (33%)	

Development of HO according to demographic, clinical or surgical factors

Four patients developed a post-operative hematoma in the DAA and two cases in the DLA group. One patient in the DLA group suffered from persistent pain after developing HO Brooker 3 and underwent surgical excision of the HO 8 month after the index THA, improving the HHS from 74 to 86. No wound complications, periprosthetic fractures, trauma, or other reasons for reoperation occurred. No medical complications delayed the rehabilitation program and ROM recovery during the follow-up period.

## Discussion

The main finding of the current study is that the DLA presents a two-times higher incidence of HO than the DAA 6 months after elective THA. The incidence of Brooker 3–4 HO was significantly higher in patients in the DLA group. Even though a lower HHS was found with severe HO, the correlation between severity of HO and clinical outcomes did not reach statistical significance. There was no significant difference in HHS of patients with HO between DLA and DAA. Except for the surgical approach, no other factors correlated with the occurrence of HO.

In the last decades, minimally invasive approaches for THA have become popular to allow the faster and less painful recovery. Less muscle damage has been associated to the DAA with better clinical outcomes in the early post-operative period and a shorter hospital stay [12, 15, 18, 19]. Conversely, the DLA induces direct muscle damage given the partial detachment of the gluteus muscles. However, the actual invasiveness of DAA is still debated, and both serum marker and imaging studies have been recently performed to quantify the invasiveness of the approach [13, 20]. Furthermore, surgeons may find that the DAA is associated with more difficult exposure and preparation of the femur with longer operative time and prolonged use of retractors, especially during the learning curve. The careless use of retractors has been proposed to cause damage muscles (e.g. TFL) in DAA and enhance the inflammatory cascade leading to HO formation. In this regard, the use of a dedicated orthopaedic table may help surgeons during the procedure, allowing for a lesser soft tissue damage to femoral exposure and a lesser muscle traction due to Homann retractors. At the same time the use of a dedicated orthopaedic table may impair surgeons in the direct assessment of leg length and implant stability [20].

In this scenario, evaluating HO development with different hip approaches might help to better clarify their invasiveness [21]. The incidence of HO after THA varies between 5 and 40% [22, 23–26]. HO usually develop within the third post-operative month. Predisposing risk factors for developing HO include male sex, previous hip surgery or trauma, a history of HO in other sites of the body, pre-existing inflammatory conditions, spinal cord injury, and surgical procedure related factors (e.g. duration of operation, blood transfusion, post-operative analgesic drugs and higher soft tissue injury) [27–32]. The pathophysiology of HO is not fully established. Local and systemic inducing agents (e.g. bone morphogenetic protein 4, prostaglandin E2, and interleukin 1) are released after surgery-related trauma. These molecules and the changes in tissue environment (e.g. hypoxia and alkalosis) may stimulate the differentiation of quiescent pluripotent mesenchymal stem cells into osteoblast within the surrounding soft tissues [3]. By limiting the surgical trauma, using a minimally invasive approach, the cascade which leads to HO development may be limited.

Regarding the role of surgical approaches, a similar incidence of HO between DAA and posterior approach (PA) was found in a retrospective study [33] on 335 patients. The HO incidence with the DAA was 24%, and was higher than that found in the present investigation. The mean operative time of their series was 144 ± 40 min, again higher than the average duration in our setting. This could partially explain the higher incidence of HO reported in that study. Furthermore, in that study the DAA was performed using a dedicated orthopaedic table which should limit the surgical soft tissue damage required to expose the femur [34].

Conversely, Zran et al. [20] investigated the incidence of HO in the DAA without a dedicated orthopaedic table. They found a significantly higher incidence of HO after DAA than after PA (47.7% vs. 27.6%, respectively). Even though this is not a comparative study between normal and dedicated orthopaedic table in DAA, the Authors of the current study found that the occurrence of HO without a dedicated orthopaedic table is not so high as reported in other previous studies and the role of a dedicated orthopaedic table could be questioned with respect to the occurrence of HO. We are not able to explain why the incidence of HO reported in those previous studies was so much higher. We have hypothesized that possible bias present in such studies (e.g. insufficient learning curve, improvement in surgical technique and instrumentation during the last few years, different surgeons involved in those studies, different post-operative pain protocols) may explain such a difference [9].

In this regard, DLA e DAA were compared by Alijanipour et al. [35] in a retrospective study of 1482 patients. A significantly higher incidence of low-grade HO was evident in the DLA group but no difference in high-grade HO was reported. However, that study presented several limitations we have tried to overcome in the current study. Firstly, three different surgeons in three different centers participated in the study. The results might have been affected by the difference in habits, technique, operative time, and peri-operative medications (i.e. a selective cox-II inhibitors and/or non steroidal anti-inflammatory drugs for pain control). Furthermore, the DLA and DAA groups were not homogeneous in term of patients’ demographics. Lastly, residents and fellows performed several cases of their series, further contributing to possible bias.

HO have been associated with functional impairment and pain [36]. In the current study, one patient in the DLA group suffered from persistent pain after HO Brooker 3 development and underwent surgical removal of the HO 8 month after THA. Even though lower HHS was present in severe HO, the difference was not statistically significant. Furthermore, even though a lower HSS was evident in HO patients in the DLA group, the difference was not statistically significant. This confirms that most of HO are asymptomatic [1] and that there is no direct association between symptoms and severity of HO. Similarly, Alijanipour et al. [34] did not find any significant difference in symptoms between Brooker 2 and Brooker 3 HO. Nevertheless, the clinical relevance of HO is still unclear and the exact impact on patients’ satisfaction need to be further elucidate. [37]All patients were operated by the same experienced surgeon with the same type of prosthesis, received the same anesthetic and analgesic protocol and were discharged with the same rehabilitation program. This might allow to minimize major important biases related to patients’ recruitment found in other retrospective or multi-center studies, and this represents a major strength of the present study. Furthermore, an independent blinded observer performed the radiographic analysis, and this contributes to a more robust interpretation of the results. Lastly, clinical evaluation established the clinical relevance of HO.

This study also presents some limitations. No prophylactic drugs for HO were used. Oral celecoxib may reduce the incidence of HO in THA, and its use is now widespread post-operatively. Patients who received post-operative celecoxib were 4.53 times less likely to develop HO than those who did not receive the medication [38–40]. This could represent both a strength and a limitation of the study at the same time. Not using prophylactic drugs, a possible confounding factor has been eliminated and the occurrence of HO is directly associated to the surgical approach. At the same time, it should be considered that the incidence of HO could be lower than that reported for both the approaches if prophylactic drugs were used. Secondly, we are aware that the study population is limited. However, being a single-center prospective study with strict inclusion and exclusion criteria which aimed to eliminate possible confounding factors, this is only a partial limitation. Lastly, we are aware that the strongest level of evidence would have been given by a randomized controlled trial. Unfortunately, this was not possible in our setting, and a quasi-randomized study was instead undertaken. However, the surgical technique, post-operative rehabilitation regimen, and the post-operative assessments were performed in a fully standardized fashion, and the analysis was undertaken in a strict scientific manner.

## Conclusions

The DAA without a dedicated orthopaedic table was associated with a significant lower incidence of HO compared to the DLA 6 months after elective THA. The surgical approach was the only factor which significantly correlated with the occurrence of HO in the two homogeneous demographic groups of patients. Even though a lower HHS was present in patients with severe HO, there was no significant association between clinical outcomes and severity of HO. Further investigations will help to elucidate the clinical relevance of HO after THA and the need actual for strategies (e.g. surgical approach and/or dedicated table and instruments) to prevent their occurrence.

## Electronic supplementary material

Below is the link to the electronic supplementary material.

Supplementary Material 1
