# Supplementary material for: “Heterotopic ossification after total hip arthroplasty through direct anterior approach without a dedicated orthopaedic table or direct lateral approach: a quasi-randomized single-center study”
Source: Arch Orthop Trauma Surg. 2024 Sep 23;144(9):4225–32. doi: 10.1007/s00402-024-05510-3 (PMC11564372; doi:10.1007/s00402-024-05510-3)
Supplement: Supplementary file 1 — Supplementary Material 1 [file 402_2024_5510_MOESM1_ESM.docx]

Dear Editor,

my coauthors and I submitted the enclosed manuscript: “Heterotopic Ossification after Total Hip Arthroplasty through Direct Anterior Approach without a dedicated orthopaedic table or Direct Lateral Approach: a quasi-randomized single-center study” for publication in your prestigious journal.
This manuscript is original and not published elsewhere. All authors have contributed significantly and all authors are in agreement with the content of the manuscript. All authors declare that there is not any conflict of interest.
The authors have no relevant financial or non-financial interests to disclose.
The authors have no competing interests to declare that are relevant to the content of this article.
All authors certify that they have no affiliations with or involvement in any organization or entity with any financial interest or non-financial interest in the subject matter or materials discussed in this manuscript.
The authors have no financial or proprietary interests in any material discussed in this article.
The authors did not receive support from any organization for the submitted work.
No funding was received to assist with the preparation of this manuscript.
No funding was received for conducting this study.
No funds, grants, or other support was received.

We hope that this study could be suitable for Your prestigious journal.
Furthermore, with the hope you’ll appreciate, our paper has been professionally proofread.

Very sincerely yours,
Edoardo Viglietta.
